# Autoantigens PLA2R and THSD7A in membranous nephropathy share a common epitope motif in the N-terminal domain

**DOI:** 10.1016/j.jaut.2019.102308

**Published:** 2020-01

**Authors:** M. Fresquet, S.J. Rhoden, T.A. Jowitt, E.A. McKenzie, I. Roberts, R. Lennon, P.E. Brenchley

**Affiliations:** a Wellcome Centre for Cell-Matrix Research, Faculty of Biology, Medicine and Health, University of Manchester, UK; b Manchester Institute of Biotechnology, University of Manchester, UK; c John Radcliffe Hospital, Oxford, UK; d Manchester University NHS Foundation Trust, Manchester Academic Health Science Centre, Manchester, UK

**Keywords:** Autoimmunity, THSD7A, PLA2R, Membranous nephropathy

## Abstract

Patients with membranous nephropathy have autoantibodies against PLA2R (up to 80%), or THSD7A (up to 2%). We previously described the immunodominant epitope within PLA2R but epitopes in THSD7A are still unknown.

To find anti-THSD7A sera for this study, we screened 1843 sera from biopsy-proven MN patients by ELISA and identified 22 sera as anti-THSD7A positive representing 1.2% of MN cases. Anti-THSD7A positive sera were further characterized by western blotting and slot blotting on THSD7A protein fragments and peptides. Real time interaction analyses and antibodies off-rate could be reliably determined using bio-layer interferometry.

A signature motif in the N-terminal domain of THSD7A (T28mer) with sequence homology to the major PLA2R epitope (P28mer) was identified. B-cell epitope prediction analysis and homology modelling revealed this sequence to be antigenic and surface available suggesting it is accessible for the antibody to bind. All ten selected sera bound to the T28mer confirming this sequence as a dominant epitope in THSD7A. Reactivity to this sequence was lost following kallikrein protease cleavage within the predicted epitope. Importantly, cross-reactivity of both PLA2R and THSD7A autoantibodies was observed at the peptide but not the protein level.

We propose that this common motif shared by both autoantigens could be an epitope involved in the initial B-cell triggering event in MN.

## Highlights

• Membranous nephropathy (MN) is an autoimmune disease characterized by the presence of autoantibodies to podocyte receptors.
• The two main antigens are PLA2R in 80% of patients and THSD7A in approximately 2% of the cases.
• A novel epitope sequence in THSD7A has high similarities with the PLA2R dominant epitope.
• This shared motif is a potential common initiating epitope of autoimmunity in MN.

## 1 Introduction

In 2009, PLA2R was defined as the predominant autoantigen in 72% of primary membranous nephropathy (MN) cases by western blotting studies using patient serum autoantibodies [1]. Subsequent clinical studies in larger MN groups, which also characterized the PLA2R antigen in immune complexes from renal biopsy samples in seronegative cases, report that PLA2R accounts for up to 80% of cases. The first Genome Wide Association Study (GWAS) in MN confirmed the importance of PLA2R as a risk factor for MN using an independent genetic strategy [2]. The major dominant epitope on PLA2R is located in the N-terminal cysteine rich domain (CysR) and its composition is a 31 amino acids peptide with a disulphide looped structure [3]. Other studies have located additional epitopes in PLA2R in particular in the C-type lectin domains 1 and 7 (CTLD1 and CTLD7) [4,5]. Current evidence suggests that the N-terminal CysR epitope is the first epitope recognized by the immune system and over time, other domain epitopes become involved in a process of epitope spreading [5].

THSD7A is defined as the second autoantigen in MN using a similar methodological approach [6] but only accounts for a small amount (up to 2%) of MN cases. No genetic evidence to support THSD7A as the second autoantigen was found in the first GWAS on 556 cases or in a recent larger GWAS on 3784 cases (Kirkluk et al. submitted 2019, personal communication). Recently, a major epitope in THSD7A was located to the N-terminal region of THSD7A with other epitopes scattered throughout the extracellular domains [7].

PLA2R and THSD7A share a number of physiochemical characteristics despite aligning to different structural protein families; they are both large N-glycosylated transmembrane receptors on the podocyte cell membrane and are composed of multiple domains which are maintained by complex patterns of disulphide bonding [8]. The main function of both receptors remains elusive but both share a single caspase-1 enzyme cleavage site in the extracellular domain, which could release an N-terminal fragment from the podocyte to form a pool of soluble antigen receptor.

MN is a rare disease with significant HLA Class II genetic restriction and yet with two autoantigens described, it displays a remarkably consistent immunopathology based on deposition of autoantibodies in the subepithelial side of the glomerular basement membrane (GBM), a similar clinical presentation and response to therapy [9].

To date, the understanding of PLA2R and THSD7A as autoantigens is that these are discrete and separate immune responses that lead eventually to a common pathology [10]. However, nothing is known about what triggers the autoantibody production. We sought evidence of an epitope motif common to both PLA2R and THSD7A autoantigens that might be revealed during antigen processing and potentially be a dominant controlling factor of antibody initiation during development of autoimmunity in MN.

## 2 Material and methods

### 2.1 Patient sera

Anti-THSD7A positive cases are very rare and therefore patient sera from various MN biobanks (n = 1843) were screened by ELISA for antibodies reactive to THSD7A and number of positive cases is shown in Table 1. Samples were obtained from the following biobanks; MRC-KRUK National DNA/Serum Bank for Glomerulonephritis, Oxford Multicentre Research Ethics Committee, UK; AUTO-MN BioBank, UK National study of Autoimmunity in MN, Research Ethics Committee 12/SW/0289; Manchester Renal Biobank, UK, Research Ethics Committee 10/H1008/10 and 16/NW/0119; MENTOR BioBank; and Toronto GN BioBank, samples were collected under the MENTOR Trial Protocol (Clinical Trials.gov NCT 01180036) with all applicable regulatory requirements; Nijmegen GN BioBank, stored biobank samples from MN patients from the Radboud University Medical Centre, samples were collected after informed consent with research authorization provided by the ethics committees of the participating centers.

**Table 1:** Source of MN cases.

Sample	Region	No.	Anti-THSD7A + ve
MRC DNA Bank	UK	320	4
AUTO-MN	UK	816	6
MENTOR	North America	162	1
Nijmegen	Netherlands	126	3
Toronto	Canada	269	5
Manchester	UK	150	3
Total		1843	22

### 2.2 Cloning, expression and purification of autoantigens and fragments

#### 2.2.1 PLA2R

The codon optimized clone of human extracellular PLA2R (Alanine_20_-Serine_1397_) was cloned into a secretory eukaryotic expression plasmid and transfected into HEK 293-EBNA1 cells using Lipofectamine 2000 reagent (Invitrogen). The secreted protein was then purified using nickel affinity chromatography (GE Healthcare) as described previously [3].

#### 2.2.2 CysR domain from PLA2R

The CysR domain of PLA2R was PCR amplified with High Fidelity Q5 PCR polymerase (NEB) using the following primers; Forward 5′ cac ggatcc gcggagggcgtggcggcg 3′ and Reverse 5′ cac gaattc tca ggtgttacccttaatggtgtgcag atccttg 3’. BamHI and EcoRI restriction sites are underlined. The resulting product was ligated to pBac His-Thioredoxin (EM proprietary insect cell transfer vector) then transformed into competent DH5a *E.coli* cells (NEB). The correct sequence of the construct was confirmed on both strands by DNA sequencing (GATC) using an internal Thioredoxin specific Forward primer and a pBac2 vector specific Reverse primer. The resulting plasmid pBacHisThioredoxin: CysR was co-transfected into Sf9 cells (OET) along with linearized baculovirus DNA to generate the recombinant virus. The recombinant virus was added to 1 L culture of High 5 insect cells (grown to a density of 2 × 10^6^ cells/ml) in Express 5 media (ThermoFisher Scientific). Cells were left 72 h post infection and the cell pellet harvested by centrifugation at 2000 rpm for 10 min at 4 °C. Pellets were resuspended in 30 ml of urea lysis buffer (8 M urea, 50 mM NaCl, 20 mM Tris-HCl, pH 7.9, 5 mM Imidazole). The cell lysate was sonicated (6 × 20  s on/off cycles at 20% amplitude setting). The lysate was then clarified by centrifugation at 17,000×*g* for 30 min at 16 °C and the supernatant incubated with 7 ml of Nickel NTA beads (Qiagen) overnight. The protein was eluted and refolded as follows. The beads were packed into a disposable column (Biorad) and then washed with 10x volumes of 8 M urea lysis buffer, 10x volumes of 50 mM NaCl, 20 mM Tris-HCl, pH 7.9, 5 mM Imidazole binding buffer, 10x volumes of 50 M NaCl, 20 mM Tris-HCl, pH 7.9, 60 mM Imidazole wash buffer and finally eluted with 10 ml of 50 mM NaCl, 20 mM Tris-HCl pH 7.9, 1 M Imidazole elution buffer. The eluate was then buffer exchanged using a PD10 column (Generon) against 150 mM NaCl, 25 mM Tris-HCl pH 7.4/10% glycerol.

#### 2.2.3 THSD7A

Codon optimized DNA sequence coding for human extracellular THSD7A (Alanine_48_-Threonine_1606_) with 10xHis tag at the C-terminus was obtained from GenScript. The constructs were transfected into HEK 293-EBNA1 cells (human embryonic kidney cells; Invitrogen) using Lipofectamine 2000 reagent (Invitrogen). Stable cell lines were established and expanded after 5–6 weeks of selection. The conditioned media diluted 1:1 in 10 mM BisTris pH 7.2, 150 mM NaCl, 10 mM imidazole was loaded onto a 5 ml HisTrap excel column (GE Healthcare). The column was then washed with five column volume using the dilution buffer containing 10 mM imidazole. The bound protein was eluted using a linear gradient from 10 mM imidazole to 500 mM over 10 ml. The fractions containing the purified protein were dialyzed in 10 mM BisTris pH 7.2, 150 mM NaCl.

#### 2.2.4 TSR1 domain from THSD7A

Bacterial codon optimized gene encoding His-tagged TSR1 (Alanine_48_-Leucine_126_) domain was synthesized (Genscript) and sub-cloned into pET 100/D-TOPO vector. The construct was freshly transformed into competent Origami 2 *E.coli* cells (Novagen). Cells were grown to an OD600 of 0.6, chilled on ice and 0.2 mM IPTG added. The cells were cultured overnight in a shaker incubator (220 rpm) at 18 °C. Cells were harvested (6500×*g* at 4 °C). The resulting frozen pellet was lysed in chilled 50 mM Tris-HCl pH 8, 0.5 M NaCl, 10% glycerol, 0.1% Triton X-100, protease inhibitor cocktail (Roche) containing 100 μg/ml lysozyme for 30 min on ice. Resuspended cells were sonicated 6 × 20 s bursts at 12% setting with cooling in between and then clarified by centrifugation at 10,000×*g* for 20 min at 4 °C. Clarified lysate was then purified by TALON affinity chromatography (Takara, ClonTech) using an imidazole wash step (10 mM) and then eluted in PBS buffer containing 300 mM imidazole. Protein was further purified on Superdex 75 column run in PBS buffer.

### 2.3 Establishment of ELISA for anti-THSD7A

The ELISA for measuring anti-THSD7A titer used a similar format to that previously published for determining anti-PLA2R [11]. The purified THSD7A protein was used to coat ELISA plates at 125 ng/ml in sodium bicarbonate buffer pH 9.6 for 18 h. Plates were blocked for 1 h with SuperBlock (Thermosystems) and kept at 4 °C until use (within 3 days). Patient serum diluted 1:100 in superblock containing 0.1% Tween 20 was added in duplicate 100 μl aliquots to the ELISA plate which also contained a standard dilution series of STD1. After 2 h incubation at room temperature on a plate shaker, the plates were washed thoroughly (eight times) with PBS plus 0.1% Tween 20. Anti-human IgG-HRP conjugate (Jackson Labs) diluted 1:25,000 in Superblock was added (100 μl per well) and incubated for 2 h as before. Following washing, enzyme substrate TMB (Sigma) was added, developed for 10 min and the reaction stopped with 0.5 M H_2_SO_4_. The plates were read at 450 nm, standard curves plotted using Softmax software and values assigned to samples. Off scale samples were diluted 1:1000–1:10,000 and reanalyzed. Standard STD1 was a pool of high titer samples (n = 4) with an allocated value of 99,000 U/ml that had been centrifuged and filtered through 0.22 μm membrane, aliquoted and stored at −80 °C. For each ELISA plate, a dilution series covering the range 3000–12.3 U/ml was applied to the plate.

#### 2.3.1 Normal range

74 serum samples from healthy individuals (mean age 40 ± 10 years) were used to define the normal range. Using mean +3SD of the normal range, we report a threshold above 66 U/ml as positive.

#### 2.3.2 Quality control

Variation between plates was measured by incorporating an aliquot diluted 1:20 of the STD1 sample in duplicate on each plate. Coefficient of variation between plates was 12%.

### 2.4 Western blotting and slot blotting

PLA2R, CysR domain, THSD7A (R&D Systems) and TSR1 protein samples (1 μg per lane) were resolved using 4–12% BisTris gradient gels with the MES buffer system, transferred to nitrocellulose membranes and blotted using patient sera (1:100 dilution) followed by incubation with Alexa Fluor® 680-AffiniPure Goat Anti-Human IgG, Fcγ Fragment Specific (min X Bov,Hrs,Ms Sr Prot) (1:10,000 dilution; Jackson Labs). Proteins were also analyzed using Moab 20-2-6, a monoclonal anti-PLA2R mouse antibody raised against a PLA2R fragment (see 2.4.1 for further details) at dilution 1:5000. Protein bands were visualized using an Odyssey Imaging System (LI-COR) at 700 nm and the intensity settings were kept constant for all blots allowing comparison. For slot blotting, 1 μg of proteins or peptides (P28mer aa38–65 and T28mer aa75-102 synthesized by ProImmune Ltd, Oxford, UK) were blotted directly onto nitrocellulose membrane and vacuum applied. For the reducing condition, samples were diluted in 10 mM Tris pH 8 then reduced and alkylated by sequential incubation with 50 mM DTT for 1 h at 37 °C and then with 125 mM iodoacetamide for 20 min in the dark. The reduced samples along with unreduced were blotted as described above.

#### 2.4.1 Generation of the mouse monoclonal antibody moab 20-2-6

Hybridoma production was undertaken by Proteogenix SAS (Schiltigheim, France). Mice were immunized with a fragment of human PLA2R protein (namely NC3, comprising the first five extracellular domains, CysR-FnII-CTLD1-CTLD2-CTLD3). Following fusion of spleen cells, 34 positive supernatants were found when screened by ELISA on the NC3 protein. Ten of the original 34 positive supernatants were taken forward for cloning. Following subcloning, 5 of 10 clones were selected for purification of monoclonal antibody which included antibody 20-2-6. Antibody 20-2-6 was selected based on strong ELISA binding to PLA2R fragment NC3.

### 2.5 Epitope prediction analysis

The B-cell epitope prediction for THSD7A was performed using BepiPred-2.0: Sequential B-Cell Epitope Predictor, a tool part of the Immune Epitope Database (IEDB) resource [12]. The BepiPred-2.0 server predicts B-cell epitopes from a protein sequence, using a Random Forest algorithm trained on epitopes and non-epitope amino acids determined from crystal structures. The residues with scores above the threshold 0.55 were predicted to be part of an epitope.

### 2.6 KLK5 digest

iProt-Sub, a Protease Specificity Prediction server [13] were used to determine single enzymatic cleavage site in the TSR1 domain of THSD7A and cutting in the T28mer sequence. The purified TSR1 protein was incubated with recombinant human KLK5 (R&D Systems) in 10 mM BisTris-HCl, pH 7.4, containing 150 mM NaCl in a 10:1 ratio for 4 h at 37 °C. The reaction was stopped by adding 4 x SDS sample buffer. Samples were subjected to SDS-PAGE and western blotting analysis.

### 2.7 Epitope homology models

Atomic coordinates for the CysR domain of PLA2R and TSR1 domain of THSD7A were generated using the Phyre 2 web portal [14]. P28mer and T28mer sequences are highlighted in yellow on the 3D homology models. A *de novo* structure prediction for the T28mer sequence was generated using the PEP-FOLD server [15]. Only the structures containing disulphide bonded cysteine were selected and manually aligned to P28mer sequence.

### 2.8 Circular dichroism (CD) of the epitope peptides

Lyophilized peptides were reconstituted in milliQ water to a concentration of 2.5 mg/ml and diluted to 0.5 mg/ml. CD Far-UV (190–260 nm) spectra were recorded using a Jasco-810 spectropolarimeter. Measurements were taken every 0.2 nm in a 0.05-cm path length cell. Spectra were corrected for buffer absorbance and represent an average of 10 accumulations. Spectra were recorded in millidegrees and converted to mean residue ellipticities using the following equation deltaE = mdeg x (0.1 x mrw)/path x conc_mg/ml x 3298 (where P28mer mrw = 109.5, T28mer mrw = 107.9). Estimation of secondary structure content was then performed using BeStSel software program [16].

### 2.9 Real time interaction studies

#### 2.9.1 Bio-layer interferometry (BLI) binding studies

##### 2.9.1.1 MN patient sera off rate evaluation

BLI experiments were conducted using an Octet Red (ForteBio, Nottingham, UK) [17]. P28mer and T28mer were diluted to 200 μg/ml in 10 mM sodium acetate, pH 4.5, and immobilized on an Octet AR2G sensor (ligand densities of 1.2 nm and 1.4 nm respectively). Patient sera were diluted 1:100 in HEPES-T sera (10 mM HEPES, 150 mM NaCl, 0.05% Tween-20 and 0.5% of a pool of human control sera). Three control normal sera were incorporated in the running buffer in order to provide the baseline binding (i.e. normalize the binding due to any spurious interaction) and allowing quantification of binding above this background. Baseline was achieved in HEPES-T sera for 600s. Sensors were incubated in MN sera for 450s (association phase) followed by HEPES-T sera for 1200s (dissociation phase). Regeneration was performed in 10 mM NaOH with three successive incubations. The peptide immobilized sensors responses to MN patient sera were calibrated against non-specific binding of each sensor to the HEPES-T sera buffer. The data were fitted to a Hill-1 plot with the inverse k value equivalent to the off rates (k_off_). Statistical analysis was performed using two way ANOVA multiple comparison or paired *t*-test as stated in the figure legends. p values of <0.05 were considered significant.

##### 2.9.1.2 Affinity purified autoantibody analysis

2 mg of MyOne™ carboxylic acid dynabeads per condition (Invitrogen, UK) were activated using 18.75 mg/ml EDC (N-3-dimethylaminopropyl- N′-ethylcarbodiimide hydrochloride, Sigma-Aldrich). 800 μg/ml of recombinant PLA2R fragment (NC3) and peptides (P28mer, T28mer or T28mer scrambled peptide) diluted in 15 mM MES buffer pH 6 were covalently immobilized overnight at 4 °C. The conditioned beads were then deactivated using 1 M ethanolamine (Bio-Rad) for 1 h. Pooled anti-THSD7A positive MN patient sera were diluted 1:50 in PBS-T and incubated (overnight at 4 °C with agitation) with 1 mg of either blank immobilized, T28mer immobilized or T28mer scrambled peptide immobilized magnetic beads. Pooled anti-PLA2R positive MN patient sera were similarly diluted and incubated with blank immobilized, PLA2R antigen immobilized or P28mer immobilized magnetic beads. Magnetic beads from all conditions were then precipitated, the supernatants removed, and washed three times in PBS-T. Bound antibodies were eluted with 0.1 M glycine pH 2.2 and the eluates from each condition were neutralized using 1 M TrisHCl pH 8. The affinity-purified eluate from each condition was then evaluated for binding to the P28mer and T28mer by BLI. The BLI analysis was executed as stated in section 2.9.1.1. with modification of the running buffer. The buffer used was HEPES-T containing affinity purified antibodies from sera (anti-PLA2R and anti-THSD7A). Control beads (blank) were incubated with the modified running buffer and the background response was used for normalization of the binding responses observed.

#### 2.9.2 Kinetics using surface plasmon resonance (SPR)

Kinetics were determined using a ProteOn XPR36 (Bio-Rad, Watford, UK). The P28mer and T28mer peptides were diluted to 200 μg/ml in 10 mM sodium acetate, pH 4.5, and immobilized on a ProteOn GLC sensor chip using standard amine coupling chemistry. Ligand densities of 2500 RU and 1500 RU were achieved for P28mer and T28mer respectively. Moab 20-2-6 was diluted to 25, 20, 15, 10, 5 nM in HEPES-T and injected for 120 s at 70 μl/min. The injection was stopped and dissociation phase measured for 800s. Buffer only and a blank immobilized lanes were used as controls and subtracted. The GLC sensor was regenerated in 10 mM NaOH with two successive injections. Kinetics were determined by fitting to a Langmuir 1:1 interaction model.

## 3 Results

### 3.1 Identification and description of anti-THSD7A positive cases

Biopsy proven cases of MN from various centers established one of the largest MN prevalent disease cohort studied to date (Table 1). We screened 1843 cases by ELISA for the purpose of identifying THSD7A autoantibodies to study. Twenty two cases were identified as anti-THSD7A positive equating to an incidence of ~1.2%. We selected ten of the anti-THSD7A MN positive patients (referred as MN01-MN10) for detailed characterization of the autoantibody specificity based on having a minimum clinical dataset and sufficient serum to study. The clinical presentation phenotypes of these cases are described in Table 2 and comprise six females and four males with a mean age at presentation of 64 years. We obtained renal biopsy tissue where available and surplus to clinical management and stained for THSD7A antigen in 8/10 cases by immunoperoxidase using rabbit anti-THSD7A antibodies (HPA000923, Sigma Aldrich, Poole, UK). All cases were positive for THSD7A in the glomerular basement membrane immune complexes and a representative result (MN01) is shown in Supplementary Fig. 1.

**Table 2:** Details of 10 MN cases under study.

Study ID	MN01	MN02	MN03	MN04	MN05	MN06	MN07	MN08	MN09	MN10
Primary/sec MN	Secondary	Primary	Primary	Primary	Primary	Primary	Primary	Secondary	Primary	Primary
Gender	F	F	F	M	M	F	F	M	F	M
Age at presentation	49	67	76	58	66	60	33	78	82	67
ethnicity	?	Cauc	Cauc	Cauc	Cauc	Afr-Amer	Cauc	Cauc	Cauc	Cauc
proteinuria uPCR	n/a	1472	1762	1413	910	7 g/24 h	643 alb/cr	1700	1420	1070
eGFR ml/min/1.73m^2^	82	65	64	53	41	60	80	43	67	54
albumin g/L	n/a	17	15	10	17	29	12	43	15	20
anti-PLA2R u/ml	<12	<12	<12	24	39	<12	<12	<12	<12	<12
anti-THSD7A u/ml	>3000	>3000	1843	2543	413	276	228	2688	713	734

### 3.2 Comparison of THSD7A and PLA2R fragments

**Fig. 1:**
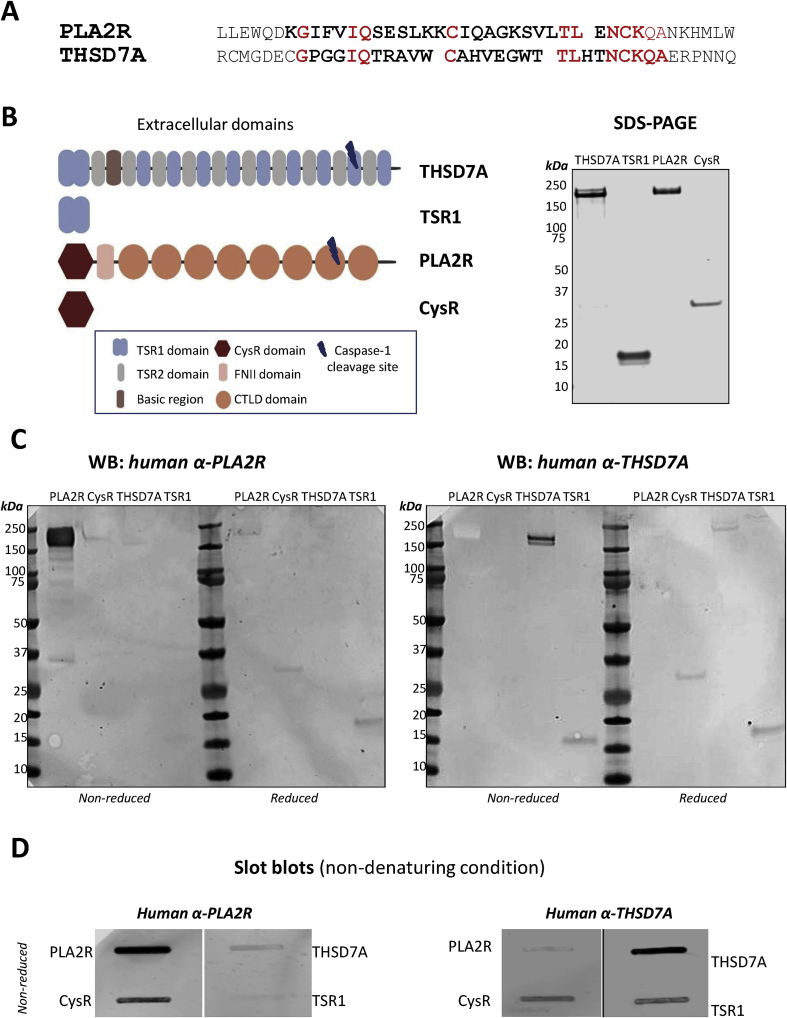
Similarities between PLA2R epitope and THSD7A. (**A**) Sequence alignment between the known major PLA2R CysR epitope peptide in bold (P28mer, aa38-65) and the potential THSD7A epitope peptide (T28mer, aa75-102) located in the TSR1 domain. Identical amino acids found across both sequences are highlighted in red. (**B**) Schematic of the extracellular domains of THSD7A comprising of 11 thrombospondin repeats (TSR), the first TSR domain (TSR1), the extracellular domains of PLA2R and the N-terminus CysR domain from PLA2R. Silver stained SDS-PAGE gel of purified recombinant THSD7A (180 kDa), TSR1 (17 kDa), PLA2R (180 kDa) and CysR (30 kDa) proteins under reducing conditions. (**C**) Western blotting analysis of denatured PLA2R, CysR, THSD7A and TSR1 proteins under non reduced and reduced conditions using a pool of five human anti-PLA2R positive sera and a pool of five anti-THSD7A sera. (**D**) Slot blotting analysis of non denatured PLA2R, CysR domain, THSD7A and TSR1 fragments under non reduced conditions. (For interpretation of the references to color in this figure legend, the reader is referred to the Web version of this article.)

We have previously shown that the N-terminal CysR domain of PLA2R contains the major B-cell epitope recognized by PLA2R autoantibodies [3]. Sequence alignment of the PLA2R CysR epitope peptide (referred as P28mer) and THSD7A revealed a peptide sequence with conserved amino acid residue positions potentially involved in the antibody binding (T28mer). We noted positional conservation of key amino acid residues in particular IQ, C, TL and NCKQA in the corresponding T28mer sequence of the first domain of THSD7A, TSR1 (shown in red, Fig. 1A). To test how common this sequence pattern might be, we searched the Uniprot/Swissprot database [18] with the PLA2R P28mer as template using only these conserved amino acids in a Position Specific Initiated BLAST search. Other than PLA2R from other species, THSD7A was the only hit showing a comparative sequence motif (Supplementary Fig. 2). The similarities between both peptides prompted us to test if the T28mer sequence is indeed an epitope in THSD7A. We analyzed the purity of the two THSD7A constructs expressing the full-length extracellular THSD7A and the first TSR domain (TSR1) and two PLA2R constructs expressing the full length extracellular PLA2R and the CysR domain (Fig. 1B). Using Western blotting we compared the reactivity of human antibodies in anti-PLA2R and anti-THSD7A positive sera against SDS denatured PLA2R, CysR, THSD7A and TSR1 fragments under reduced and non-reduced conditions (Fig. 1C, Supplementary Fig. 3). Autoantibodies in anti-PLA2R positive sera only recognized PLA2R under denatured and non-reduced conditions. Upon reduction, this reactivity was lost and faint reactivity appeared against TSR1. Autoantibodies in anti-THSD7A positive sera under denatured non-reducing conditions reacted against THSD7A and weakly to TSR1. Upon reduction, this reactivity was lost but the weak reactivity to TSR1 remained. A similar comparison of antibody reactivity to the protein fragments was performed under non-denaturing conditions (Fig. 1D). Antibodies in anti-PLA2R positive sera reacted strongly with PLA2R and CysR fragments and weakly with THSD7A and TSR1. Antibodies in anti-THSD7A positive sera reacted strongly with THSD7A and TSR1 and interestingly cross-reacted with the CysR domain. This apparent cross-reactivity is further discussed later in this study.

### 3.3 Identifying a major epitope in THSD7A

**Fig. 2:**
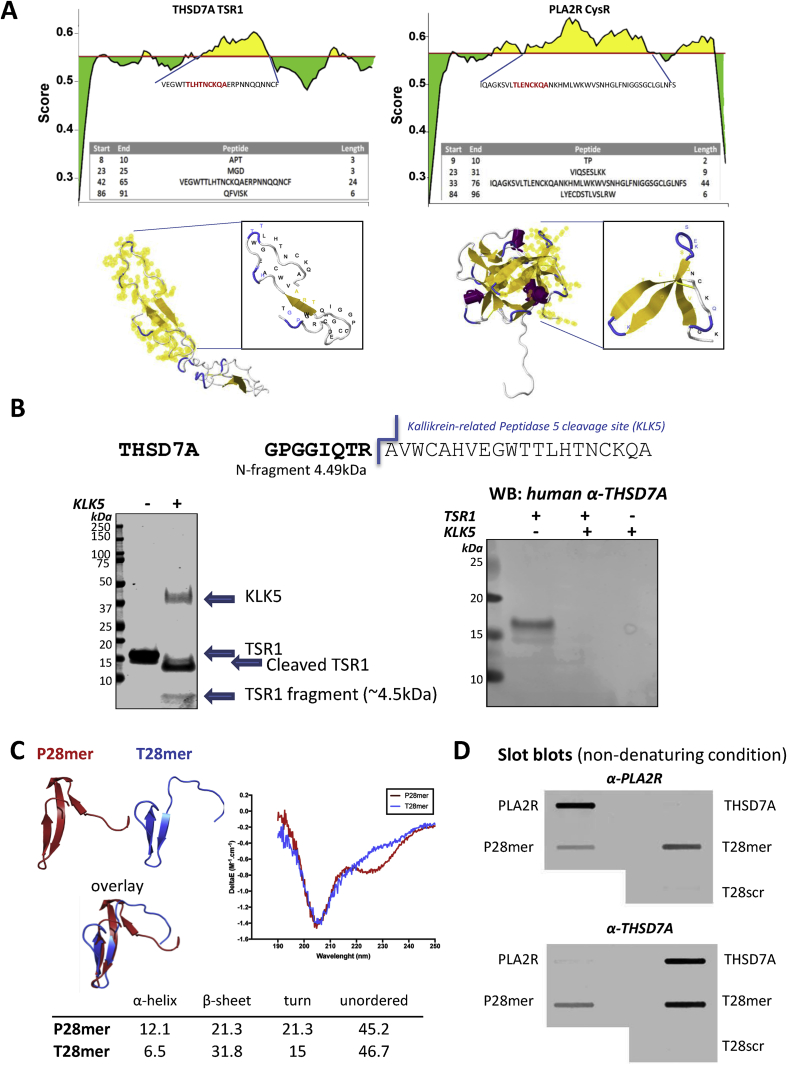
Finding the major epitope in THSD7A. (**A**) B Cell epitope prediction for THSD7A using BepiPred-2.0 software. The BepiPred-2.0 server predicts B-cell epitopes from a protein sequence, using epitope data derived from crystal structures. The residues with scores above the threshold (0.5) are predicted to be part of an epitope and colored in yellow on the graph (where Y-axes depict residue scores and X-axes residue positions in the sequence). The predicted peptides sequences are shown in the table and mapped (amino acid residues in yellow) on the homology models of TSR1 and CysR domains respectively highlighting their surface availability. (**B**) Predicted cleavage site of kallikrein-related Peptidase 5 (KLK5) shown on the THSD7A epitope sequence, releasing a 4.5 kDa N-terminus fragment. SDS-PAGE analysis of undigested (−) and digested (+) TSR1 domain with KLK5 enzyme demonstrating the fragmentation of the domain and release of the 4.5 kDa fragment. Western blotting analysis of intact and fragmented TSR1 by KLK5 digest using a pool of anti-THSD7A patient sera. (**C**) Structure of modelled P28mer (red) and T28mer (blue) peptides and their overlay. PLA2R and THSD7A sequences were subjected to 3D *de novo* structure prediction using PEP-FOLD and the best structures output as PDB coordinates. The secondary structure of P28mer and T28mer peptides was determined by circular dichroism. Superimposed CD spectra from both peptides and estimation of their secondary structure content using BeStSel software (*table*). (**D**) Slot blotting analysis of native NC8, THSD7A, P28mer, T28mer and a scrambled version of T28mer peptides (T28scr, as control) under non reducing conditions using a pool of five human anti-PLA2R and anti-THSD7A positive sera. (For interpretation of the references to color in this figure legend, the reader is referred to the Web version of this article.)

The CysR and TSR1 domain sequences were analyzed using the B-cell epitope prediction software BepiPred-2.0, which combines epitope data derived from crystal structures. Antigenicity prediction profiles were identified covering the T28mer and P28mer sequences and modelling of the domains highlighted their surface accessibility, a characteristic of B-cell epitopes (Fig. 2A).

The Protease Specificity Prediction server iProt-Sub predicted a single serine protease KLK5 cleavage site in the TSR1 domain of THSD7A, specifically in the T28mer sequence. In Fig. 2B, we show that the TSR1 domain is sensitive to KLK5 digestion cleaving the predicted 4.5 kDa fragment and that KLK5 digestion destroys the recognition of TSR1 by THSD7A autoantibodies. This confirms that the T28mer sequence represents the only epitope in the TSR1 domain recognized by THSD7A autoantibodies.

The P28mer and T28mer peptides were subjected to 3D *de novo* structure prediction using PEP-FOLD and the best structures output as PDB coordinates. Both models showed characteristics of a trefoil-like structure. The generated models were then aligned and showed partial overlay of both peptides suggesting a similar 3D structure (Fig. 2C). Both P28mer and T28mer peptides were synthesized and showed secondary structure in solution as determined by circular dichroism and overall similar profiles validating the models. The P28mer peptide displayed two negative peaks around 205 nm and 227 nm indicative of both alpha-helical and beta-sheet structures. The T28mer peptide had also a peak in the negative region of the CD spectrum around 205 nm. However, the two peptides showed slightly different composition in their alpha-helical and beta-sheet content as seen in the table (Fig. 2C).

On testing antibodies in either anti-PLA2R or anti-THSD7A positive sera binding to PLA2R, P28mer, THSD7A and T28mer by slot blotting under non-denaturing conditions, we showed at the protein level anti-PLA2R is specific to PLA2R and anti-THSD7A is specific to THSD7A. We observed no binding of anti-THSD7A to PLA2R protein or anti-PLA2R to THDS7A protein. However at the peptide level, anti-PLA2R reacted with both P28mer and T28mer and not the peptide control (Fig. 2D, Supplementary Figs. 4 and 5). Similarly, anti-THSD7A reacted with both P28mer and T28mer epitope peptides and not the peptide control. This interaction was specific to MN autoantibodies as no binding to either P28mer or T28mer could be detected using sera from two other glomerular diseases, ANCA vasculitis and IgA Nephropathy (IgAN) respectively (Supplementary Fig. 6). This is the first indication of possible cross-reactivity between the two autoantibodies and the two antigens.

These data confirm that the TSR1 domain contains a dominant epitope and demonstrate that availability within the mature THSD7A protein of the T28mer epitope to antibody binding is dependent on the local domain conformation.

### 3.4 Cross-reactivity between the two autoantibodies and their antigens

**Fig. 3:**
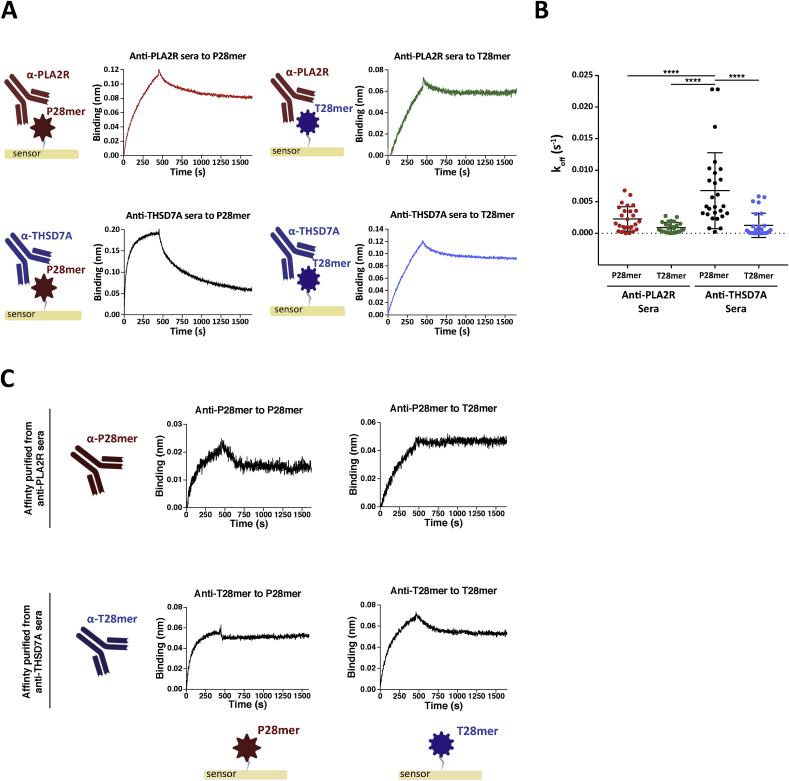
Cross-reactivity between the two autoantibodies and their antigens. (**A**) Sensorgrams of typical binding response of anti-PLA2R positive sera to either immobilized P28mer (red) or T28mer (green) (*top panel*); anti-THSD7A positive sera to either immobilized P28mer (black) or T28mer (blue) (*bottom panel*). (**B**) Quantification of the dissociation rates was analyzed by origin pro hill 2 plots for all (10) anti-PLA2R and (10) anti-THSD7A positive MN patient sera. **** Indicates p value < 0.001 (two-way ANOVA). (**C**) Sensorgrams of typical binding response of affinity purified anti-P28mer to either immobilized P28mer (left) or T28mer (right) (*top panel*); anti-T28mer to either immobilized P28mer (left) or T28mer (right) (*bottom panel*). (For interpretation of the references to color in this figure legend, the reader is referred to the Web version of this article.)

To further characterize the cross-reactivity of sera autoantibodies to T28mer and P28mer epitope peptides, we determined their rates of dissociation (off-rate) using Bio-Layer Interferometry (BLI). The P28mer and T28mer target epitopes were immobilized onto separate BLI sensors and ten different anti-THSD7A or anti-PLA2R positive sera were sequentially flown across both antigen peptides (Fig. 3). The resulting binding was normalized to a pool of three healthy control sera. Once antigen-antibody complex has formed, the off-rate is dependent on the strength of the interaction and independent of the concentration. The higher the affinity, the slower the off-rate (K_off_) value is. We determined that PLA2R antibodies within serum bound to P28mer or T28mer and formed peptide-antibody complexes with similar slow dissociation rates (K_off_ values of 2.8 × 10^−3^ s^−1^ and 0.9 × 10^−3^ s^−1^ respectively), Fig. 3A (Supplementary Figs. 7 and 8). Sera THSD7A antibodies appeared to have a preference for binding to its own T28mer epitope compared to the P28mer peptide, with dissociation rates of 1.3 × 10^−3^ s^−1^ and 6.8 × 10^−3^ s^−1^ respectively (Fig. 3A). This finding suggests that sera THSD7A autoantibodies preferentially bind to the T28mer over P28mer, whereas sera PLA2R autoantibodies show no preference in binding either peptide (Fig. 3B). To determine whether a particular antibody population within anti-PLA2R and anti-THSD7A sera is bispecific for both P28mer and T28mer peptides, PLA2R autoantibodies were affinity purified against the P28mer (anti-P28mer) and THSD7A autoantibodies against the T28mer (anti-T28mer). Anti-PLA2R positive sera were also incubated with recombinant PLA2R coated beads as a positive control. Each affinity purified antibodies were then tested for their reactivity to both P28mer and T28mer peptides immobilized on separate BLI sensors (Fig. 3C, Supplementary Fig. 9). Despite using small quantity of serum and small volume of beads, it was possible to affinity isolate enough antibodies to detect significant binding. PLA2R autoantibodies, anti-P28mer and anti-T28mer (originating from anti-THSD7A sera) were found to bind to both epitope peptides demonstrating their cross-reactivity capability.

### 3.5 A single antibody species shows similar binding specificity to both epitope peptides

**Fig. 4:**
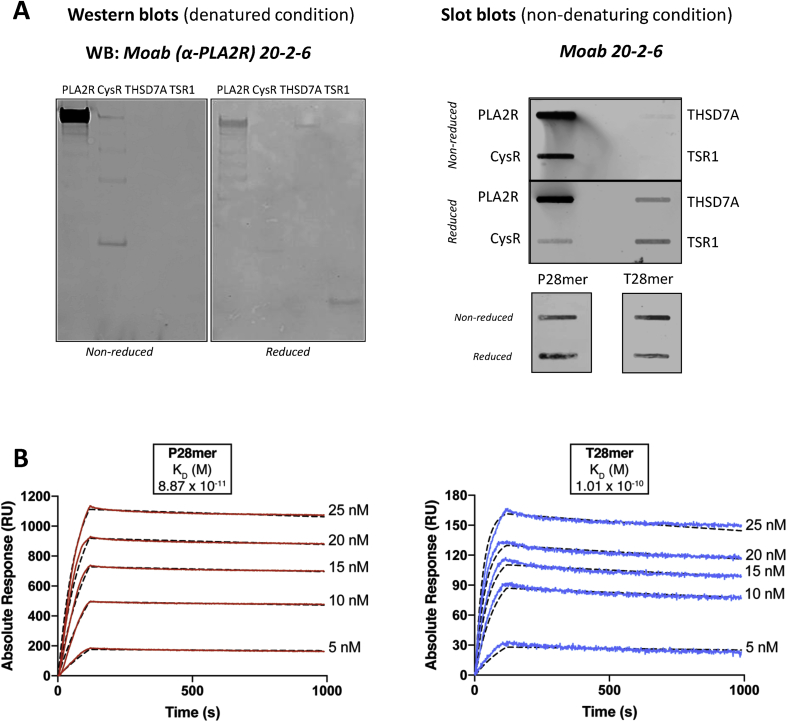
A mouse monoclonal antibody shows similar binding specificity to both epitope peptides. (**A**) Characterization of a mouse monoclonal raised against PLA2R. Western blot analysis of PLA2R and THSD7A variants using mouse Moab 20-2-6 under reducing and non reducing conditions. Slot blotting analysis of PLA2R, CysR, THSD7A and TSR1 purified proteins, P28mer and T28mer peptides reduced and non reduced incubated with Moab 20-2-6. (**B**) SPR analysis of the binding between Moab 20-2-6 and captured P28mer (*left*) or T28mer (*right*). Sensorgrams derived from injections of different concentrations of Moab 20-2-6 PLA2R-specific antibody over immobilized peptides. Kinetics data were fitted to a Langmuir 1:1 interaction model with mass transfer.

Using standard hybridoma methodology, we produced a suite of monoclonal antibodies recognizing the PLA2R fragment (comprising of the CysR domain, fibronectin type 2 domain and the first 3 C-type lectin domains) including the previously described Moab 12-6-5 [19]. Characterization of another mouse monoclonal (Moab 20-2-6) revealed reactivity by western blotting to PLA2R (full length extracellular domains) and the CysR domain under non reducing conditions; this reactivity was greatly reduced under reducing conditions. In addition the antibody did not recognize THSD7A (full length extracellular domains) or the TSR1 domain proteins under non reducing conditions, but showed some reactivity under reduced conditions (Fig. 4A, *left panel*). Native slot blotting analysis confirmed the reactivity pattern seen by western blotting (Fig. 4A, *right panel*). Specificity to both peptides P28mer and T28mer was confirmed by testing an unrelated mouse monoclonal, demonstrating no reactivity (Supplementary Fig. 10). Binding kinetics between both epitope peptides and Moab 20-2-6 confirmed a tight interaction of 8.9 × 10^−11^ M with P28mer and 1 × 10^−10^ M with T28mer (Fig. 4B). These data show that a single mouse monoclonal PLA2R antibody recognizes both P28mer and T28mer epitope peptides with high affinity, similar to the human autoantibody.

## 4 Discussion/conclusions

In this study we have described an amino acid pattern unique to the N-terminal region of the membranous nephropathy autoantigens PLA2R and THSD7A that constitutes an epitope. We present experimental data supporting a novel and unifying hypothesis that mature PLA2R and THSD7A autoantibodies recognize this sequence/shape (P28mer and T28mer) in the free peptide configuration. All ten anti-THSD7A sera tested recognized the T28mer peptide by native blotting suggesting it is a dominant epitope in the THSD7A antigen. This is in agreement with a recent report stating 84% of MN patients (n = 31) seropositive for anti-THSD7A reacted with the N-terminal TSR1 domain of THSD7A [7]. We propose that this dominant epitope, which is recognized by both mature PLA2R and THSD7A autoantibodies in the peptide form but not in mature protein antigen form, could represent the initial epitope fragment that binds to and triggers the Ig receptor on B cells. Understanding the relevance of this cross-reactive epitope binding may provide an explanation of how autoantibodies to PLA2R and THSD7A are generated by B cells in patients with MN.

The specificity of the mature immune response to PLA2R and THSD7A may contain clues as to how it originated, but the specificity will be different from the early response. However, identifying MN patients at the early stage of the disease is currently impossible. MN is characterized as a chronic lesion; once the immune response has initiated and autoantibodies are produced, the patient is unaware of the disease onset until proteinuria occurs causing symptoms of frothy urine or oedema. So the time from first autoantibody production to disease diagnosis by biopsy may be months to years. This lengthy timeline is supported by the maturity of the autoantibody response at presentation. Class switching to predominantly IgG4 has already occurred, somatic mutation has facilitated the maturation of the antibody to high affinity and other epitopes are detectable.

We confirmed the presence of cross-reactivity between the major epitope in PLA2R (P28mer) and the similar epitope in THSD7A (T28mer) at an early stage in antibody production by raising a monoclonal antibody against the PLA2R epitope. Cloning the antibody as part of the hybridoma methodology ensures it is a single clonal IgG species (and not a polyclonal antibody) that is able to bind to both epitopes. Using synthetic peptide epitopes P28mer and T28mer ensures the target is well defined. A single antibody species binding to both peptides proves the cross-reactivity. This antibody retained specificity for PLA2R at the protein level implying the epitope in PLA2R is surface available and in a correct conformation whereas in THSD7A this may not be the case. This effect may also contribute to the observed specificity of patient PLA2R and THSD7A autoantibodies for the respective protein. However, in the testing of patient autoantibodies for specificity on protein targets, additional factors control operational specificity, in particular where additional antibodies to other epitopes unique to that protein are present as part of the maturing polyclonal response. Interestingly both P28mer and T28mer peptides contain a C-terminal sequence (SVLTLENCKQA and TLHTNCKQA respectively) which shares similarity with the previously described sequence LTLENCK found in *Clostridium* peptidase S11 [3]. Other organisms such as S*accharomyces cerevisiae* and *Pseudomonas HrcC Type III* also contain an amino acid sequence (SVLTLEN) which overlap with the epitope motif. This raises the possibility that exposure to microbes during the development of natural immunity in early life may set the foundation for a role in the initiation of MN in middle age. Pathogen exposure is linked with several autoimmune diseases [20] and molecular mimicry [21] where common or similar amino acid sequences between microorganisms and host is a possible mechanism of initiation of autoimmunity. Indeed microbial antigen exposure could increase the frequency of IgM positive B cells that have the ability to bind part of the P28mer/T28mer epitope. However, for autoimmunity to be triggered to produce IgG autoantibody, the autoantigen PLA2R or THSD7A must be processed and peptides presented in a genetically restricted class II receptor to T cells (only possible by patients at risk of MN). This genetic risk for MN, embedded in HLA DQA1 and PLA2R genes [2], is likely mitigated through the protein products of these genes presenting autoantigen peptide to T cells. This interaction provides T cell help to B cells which have been triggered by autoantigen epitope fragments binding the B cell Ig receptor. The B cells can then differentiate into plasma cells secreting IgG antibody which in the early response is able to bind PLA2R and THSD7A. The amino acid composition/structure of the two antigen epitopes P28mer and T28mer are similar enough in shape that the antibody fits them both, triggering the immune attack.

**Fig. 5:**
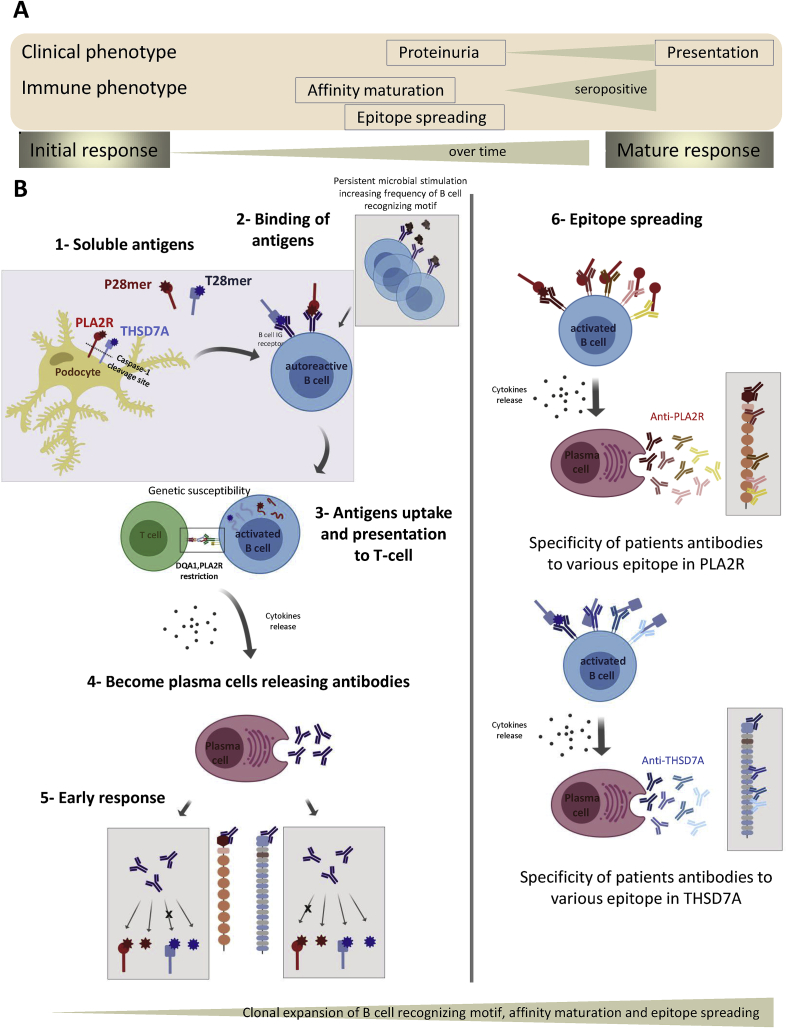
Potential mechanism for initiation of MN and autoantibody cross-reactivity. (**A**) Development of clinical and immune phenotype. Dissociation in time between primary immune response to PLA2R and clinical phenotype (from proteinuria to diagnosis of MN disease). The likely time difference between the initial immune processing of PLA2R to generate the primary immune response and classification of MN with anti-PLA2R seropositivity is months to years. Only the matured response, post affinity maturation, post subclass switching, post epitope spreading is present at disease detection. (**B**) Caspase-1 cleavage site in the extracellular domains of PLA2R and THSD7A proteins could allow release of an N-terminal antigen fragment from the podocyte to engage with B cell receptor initiating an autoimmune response. Persistent microbial stimulation primes the B cell recognizing motif and increases the frequency of these autoreactive B cells. Presented antigen fragments (containing the P28mer or T28mer sequence) activate the B cell, uptake the antigen and present peptides to T cell, which in turn trigger cytokine release and become plasma cells releasing autoantibodies. As the response matures, many processes in the development of the autoantibody occur such as B cell proliferation, differentiation, class switching, affinity maturation and finally epitope spreading. These processes may account for the two following outcomes: 1) 80% of anti-PLA2R positive cases (anti-THSD7A negative); 2) 1.2% of anti-THSD7A positive cases (anti-PLA2R negative).

We propose a new concept of the origin of PLA2R and THSD7A autoantibodies (Fig. 5). The implication of our finding is that either PLA2R or THSD7A antigens, when fragmented and processed by the immune system, release the epitope P28mer or T28mer peptides that bind the Ig receptor on the B cell initiating the immune reaction. At this early stage the B cell receptors are poly-reactive, of low affinity and capable of binding diverse epitope structures. However persistent stimulation with antigen peptide over time drives affinity maturation of the antibody and also induces the Ig class switch from IgM through IgG1/G3 to IgG4 (Fig. 5). In addition to these events, the mature polyclonal response to PLA2R and THSD7A will include autoantibodies to several other epitopes in each protein in addition to the initiating epitope, which will convey specificity to either PLA2R or THSD7A in the majority of patients. This is exactly what is described for the majority of patients at presentation who are either anti-PLA2R positive or anti-THSD7A positive when screened against the autoantigen proteins by WB, ELISA or immunofluorescence. Interestingly, one study [22] identified two MN cases as dual positive when renal biopsies were immunostained (i.e. both antigens were apparently present in the GBM immune complexes) but as no serology was reported in these cases the specificity/cross-reactivity of the autoantibodies is unknown. Another report confirmed two Asian [23] patients as dual positive by renal biopsy staining and serology and with no evidence of cross-reactivity of the autoantibodies on protein antigens but they did not examine cross-reactivity on peptide epitopes. A recent study from a large patient cohort identifies eight dual positive cases (includes the four mentioned cases) but again they did not test for cross-reactivity on peptide epitopes [24].

These reports on extremely rare cases of apparent dual protein positivity illustrate the clonal diversity that the immune system is able to generate over time, but do not negate the idea that a common epitope motif in both autoantigens initiated the primary autoantibody response.

We present the first evidence that there is a common dominant B cell epitope in these two autoantigens that could potentially be at the heart of the autoimmune aetiology of MN and provides a hypothesis to test that individuals at high risk of MN or in the primary phase of autoantibody production may have circulating B cells with an Ig receptor able to bind both P28mer and T28mer peptides.

## Competing financial interests

P.B. and M.F. are inventors on patent WO 2015/185949A1 and the same authors with R.L. and S.R. are inventors on a further patent filed with UK Patent Office. The other authors declare no competing financial interests**.**
