# Supplementary material for: Autoantigens PLA2R and THSD7A in membranous nephropathy share a common epitope motif in the N-terminal domain
Source: J Autoimmun. 2020 Jan;106:102308. doi: 10.1016/j.jaut.2019.102308 (PMC7471840; doi:10.1016/j.jaut.2019.102308)
Supplement: Multimedia component 2 [file mmc2.pdf]

Supplementary information

Autoantigens PLA2R and THSD7A in membranous nephropathy share a common epitope motif in the N-terminal domain

M. Fresquet-S.J. Rhoden*, T.A. Jowitt, E.A. McKenzie, I. Roberts ,
R. Lennon, P.E. Brenchley

Supplementary Fig.1 - Kidney biopsy staining of anti-THSD7A positive patient

Supplementary Fig.2 - Position specific iterated (PSI) BLAST search of UniProtKB/Swissprot database using PLA2R motif identifies the sequence in THSD7A

Supplementary Fig.3 - Western blot analysis of ten anti-THSD7A MN patients

Supplementary Fig.4 - Slot blot analysis of ten anti-THSD7A MN patients

Supplementary Fig.5- Slot blot analysis of ten anti-PLA2R MN patients

Supplementary Fig.6 - Slot blot analysis of sera from IgAN and ANCA vasculatis patients

Supplementary Fig.7 - Sensorgrams of ten anti-THSD7A MN patients

Supplementary Fig.8 - Sensorgrams of six anti-PLA2R MN patients

Supplementary Fig.9 - Specificity of affinity purified anti-PLA2R to P28mer and T28mer

Supplementary Fig.10 - Specificity of Moab 20-2-6 to P28mer and T28mer. Slot blot analysis of unrelated mouse monoclonal

Supplemental data

Fig.1 - Kidney biopsy staining of anti-THSD7A positive patient

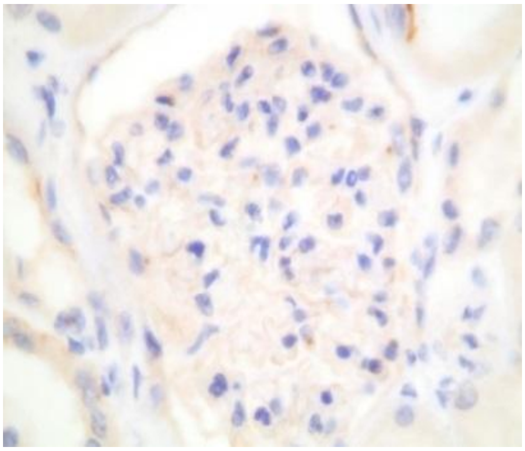

Normal Kidney x40

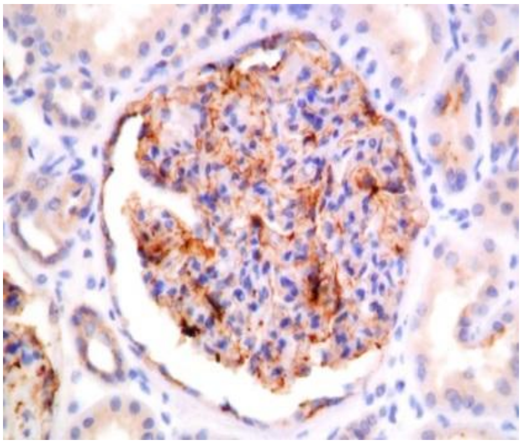

MN01 Kidney x40

Immunohistochemistry images of paraffin-embedded human kidney tissues stained with anti-THSD7A (Atlas, HPA000923). *Left*, Normal human kidney showing low background staining. *Right*, Membranous nephropathy kidney (patient MN01) showing staining of THSD7A in the capillary wall of the glomerular basement membrane. Magnification, x40.

Fig.2 - Position specific iterated (PSI) BLAST search of UniProtKB/Swissprot database using PLA2R motif (Motif) identifies the sequence in THSD7A (Match)

Alignment statistics for match #1

Score	Expect	Identities	Positives	Gaps
25.2 bits(52)	2.7	10/28(36%)	10/28(35%)	1/28(3%)

```
Motif   1    GXXXIQXXXXXXCXXXXXXXTLXXNCKQ   28
             G   IQ      C       TL  NCKQ
Match   75   GPGGIQTRAVW-CAHVEGWTTLHTNCKQ   101   THSD7A
```

Fig.3 - Western blot analysis of ten anti-THSD7A MN patients

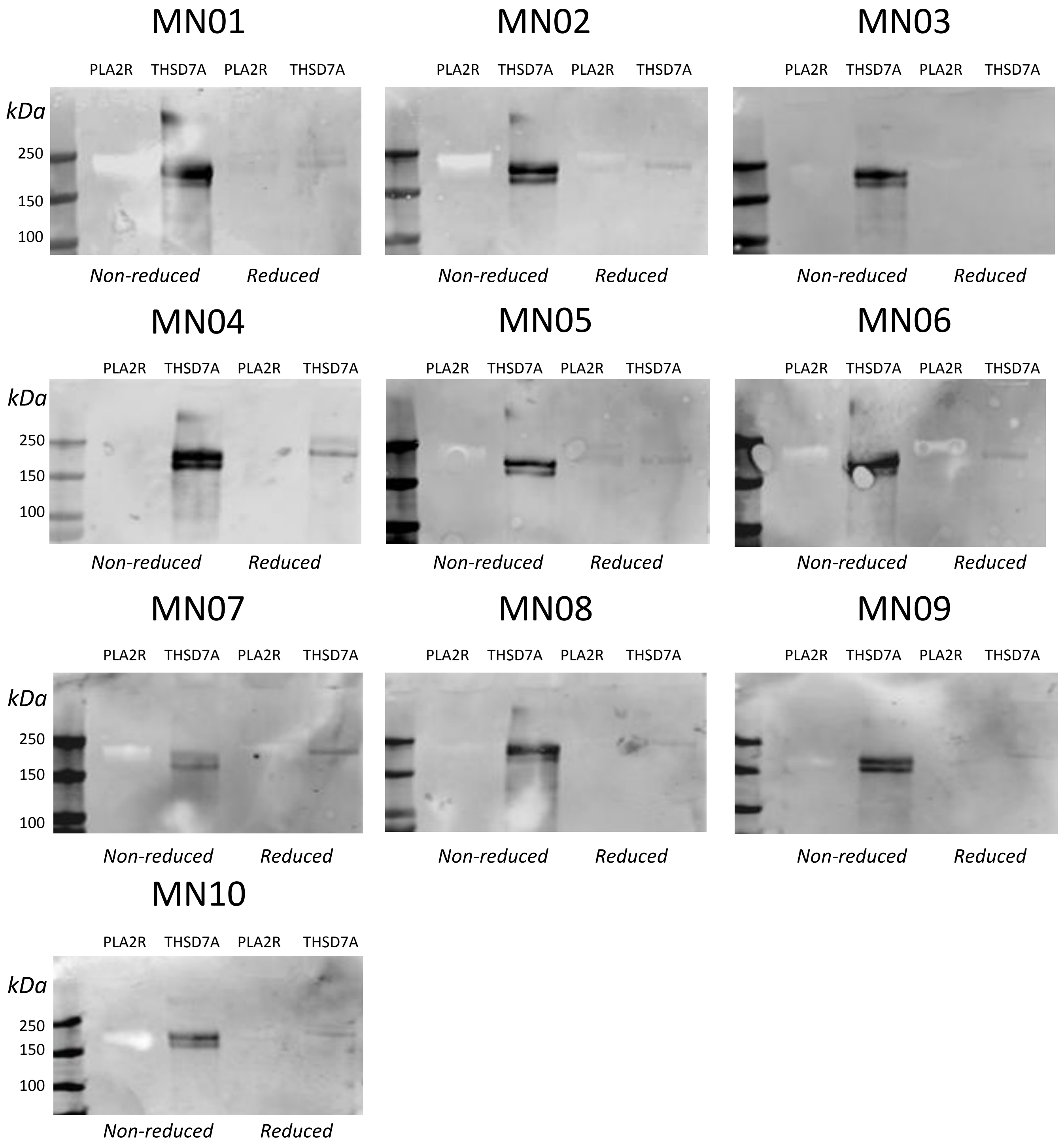

Western blot images of PLA2R and THSD7A under non-reducing and reducing conditions incubated with 10 anti-THSD7A positive patients sera. Each lane was loaded with 1µg of protein and the membrane incubated with sera diluted 1:100.

Fig.4 - Slot blot analysis of ten anti-THSD7A MN patients

***Slot blots** (non-denaturing condition)*

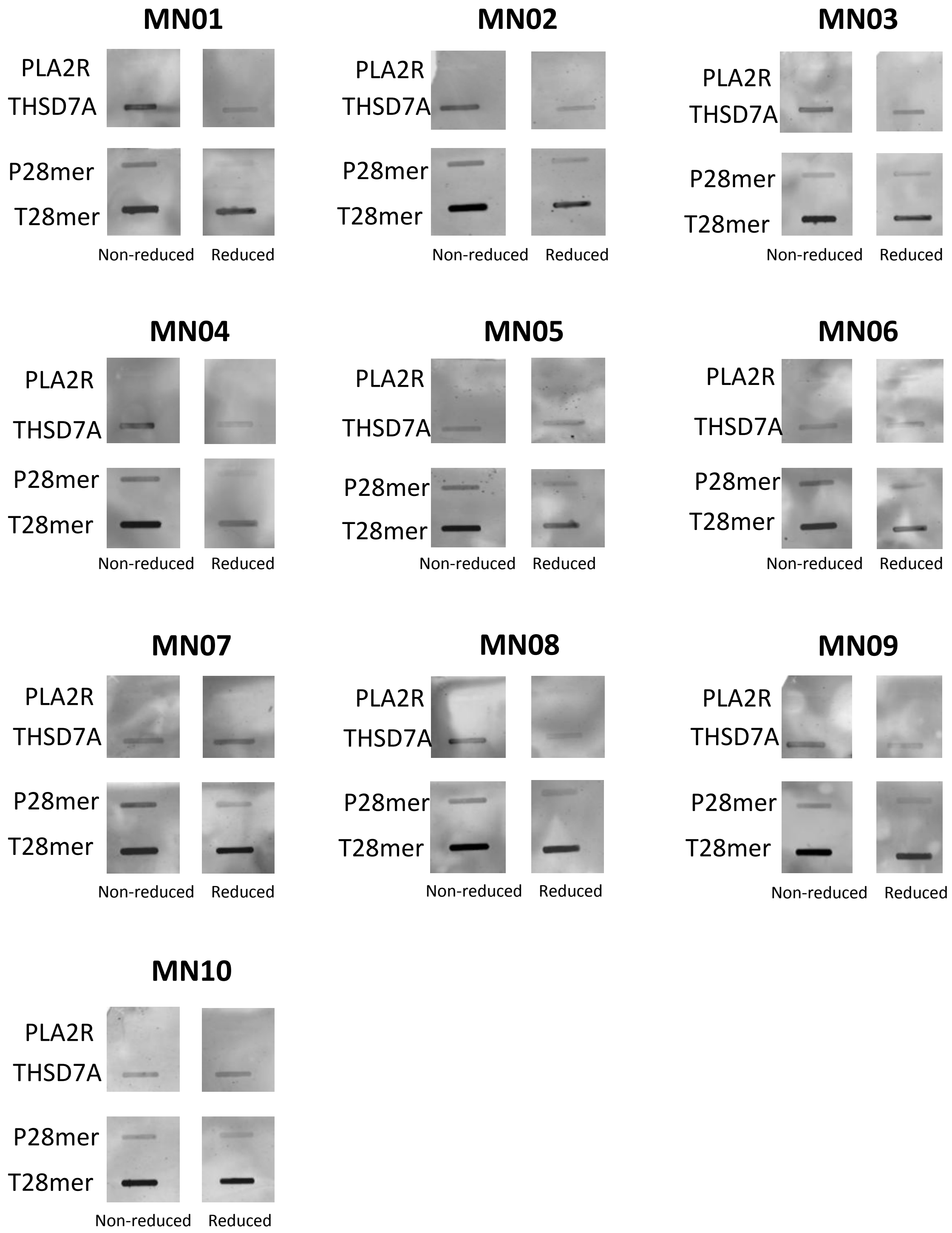

Slot blot images of PLA2R, THSD7A, P28mer and T28mer under non-reducing and reducing conditions incubated with 10 anti-THSD7A positive patients sera. Each slot was loaded with 1µg of protein/peptide and the membrane incubated with sera diluted 1:100.

Fig.5 - Slot blot analysis of ten anti-PLA2R MN patients

***Slot blots** (non-denaturing condition)*

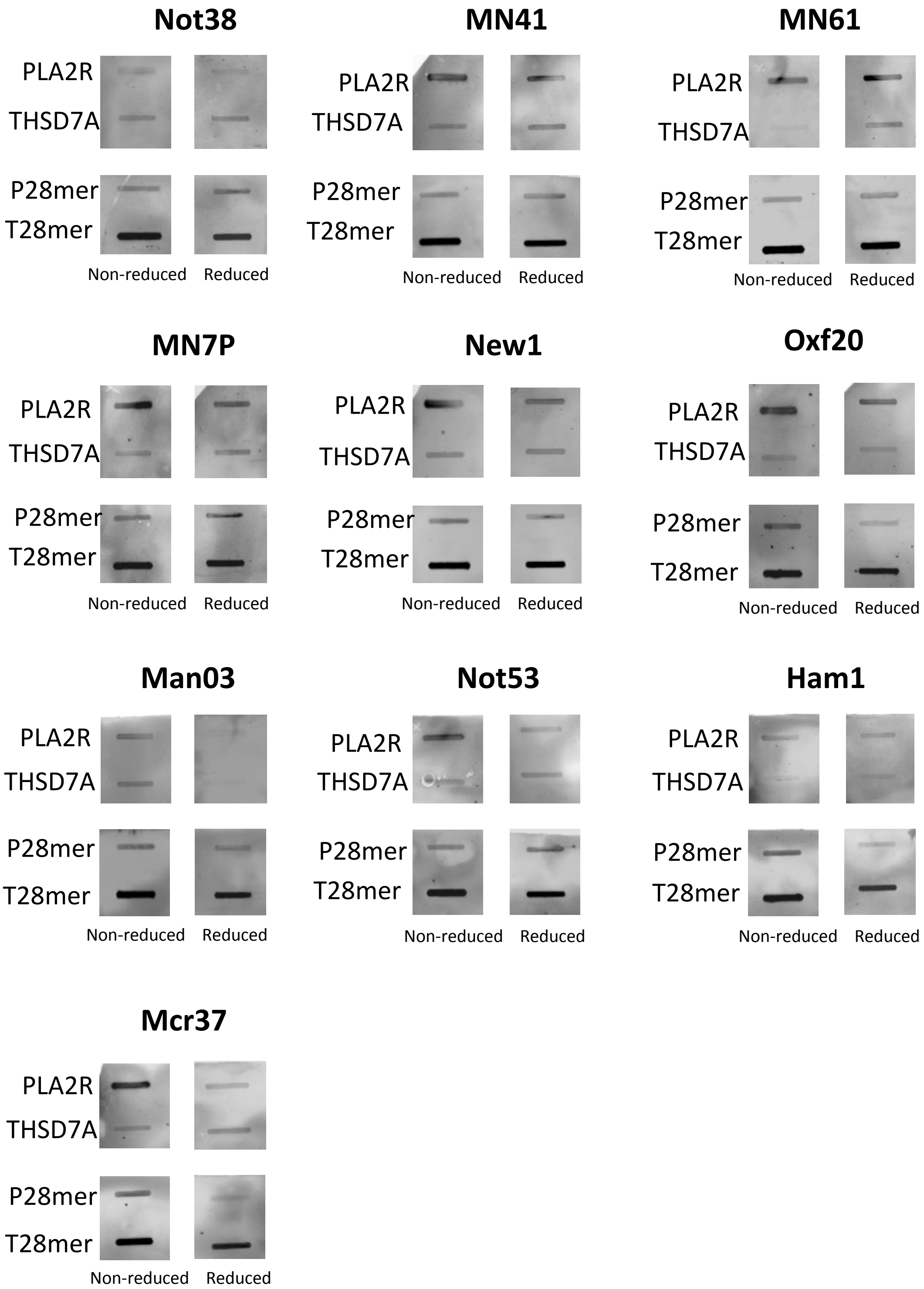

Slot blot images of PLA2R, THSD7A, P28mer and T28mer under non-reducing and reducing conditions incubated with 10 anti-PLA2R positive patients sera. Each slot was loaded with 1µg of protein/peptide and the membrane incubated with sera diluted 1:100.

Fig.6 - Slot blot analysis of sera from IgAN and ANCA vasculitis patients

Non-reduced

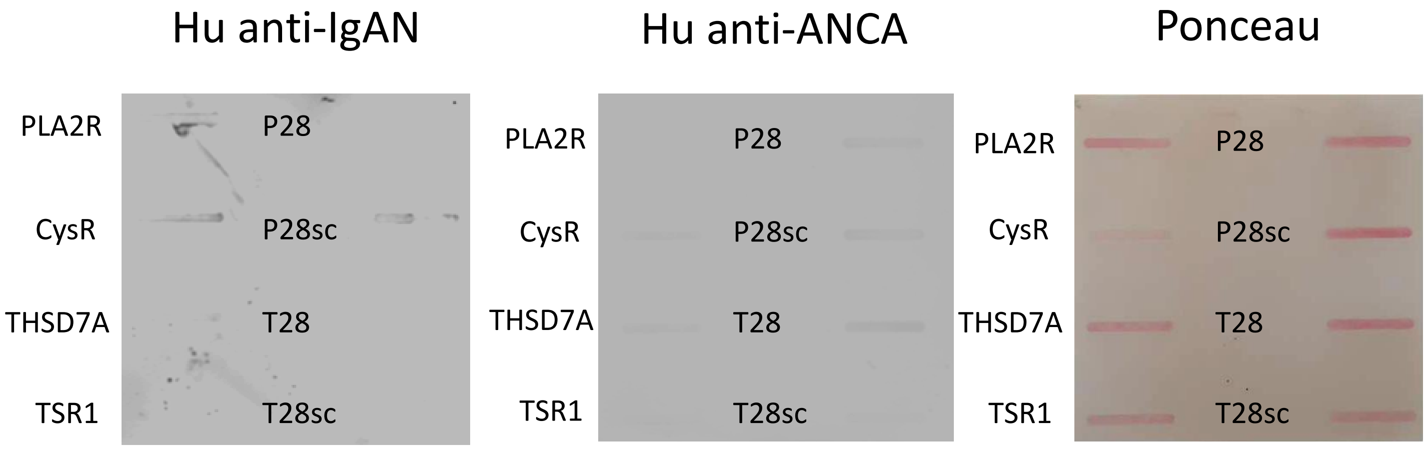

Slot blot images of PLA2R, CysR, THSD7A, TSR1, P28mer and T28mer under non-reducing conditions incubated with a pool of 5 anti-IgAn positive patients sera (left) or incubated with a pool of 5 anti-ANCA positive patients sera (right). Each slot was loaded with 1µg of protein/peptide and the membrane incubated with sera diluted 1:100.
The Ponceau staining was used to confirm protein/peptide loading onto the membrane.

Fig.7 - Sensorgrams of ten anti-THSD7A MN patients

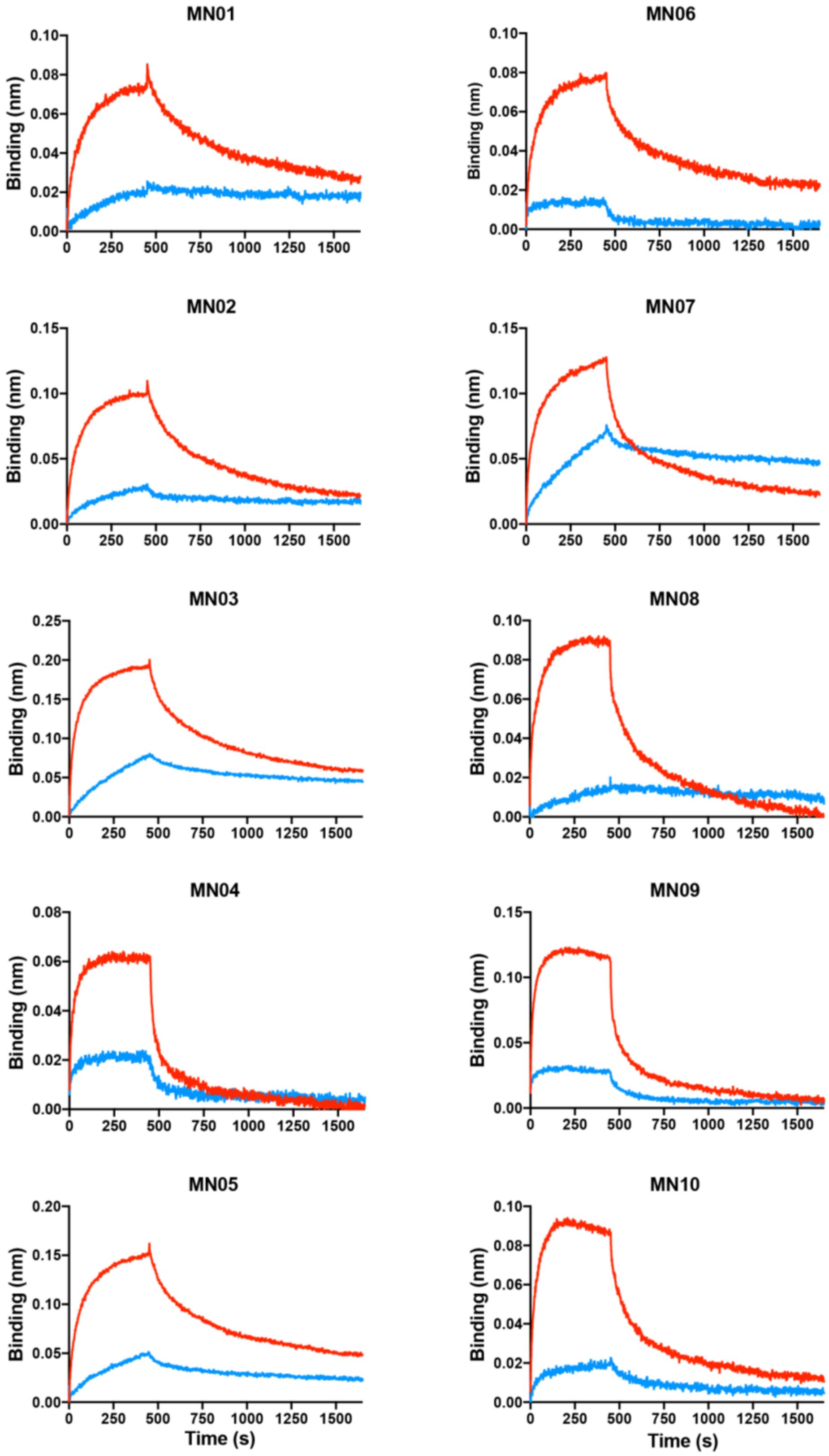

Fig.8 - Sensorgrams of ten anti-PLA2R MN patients

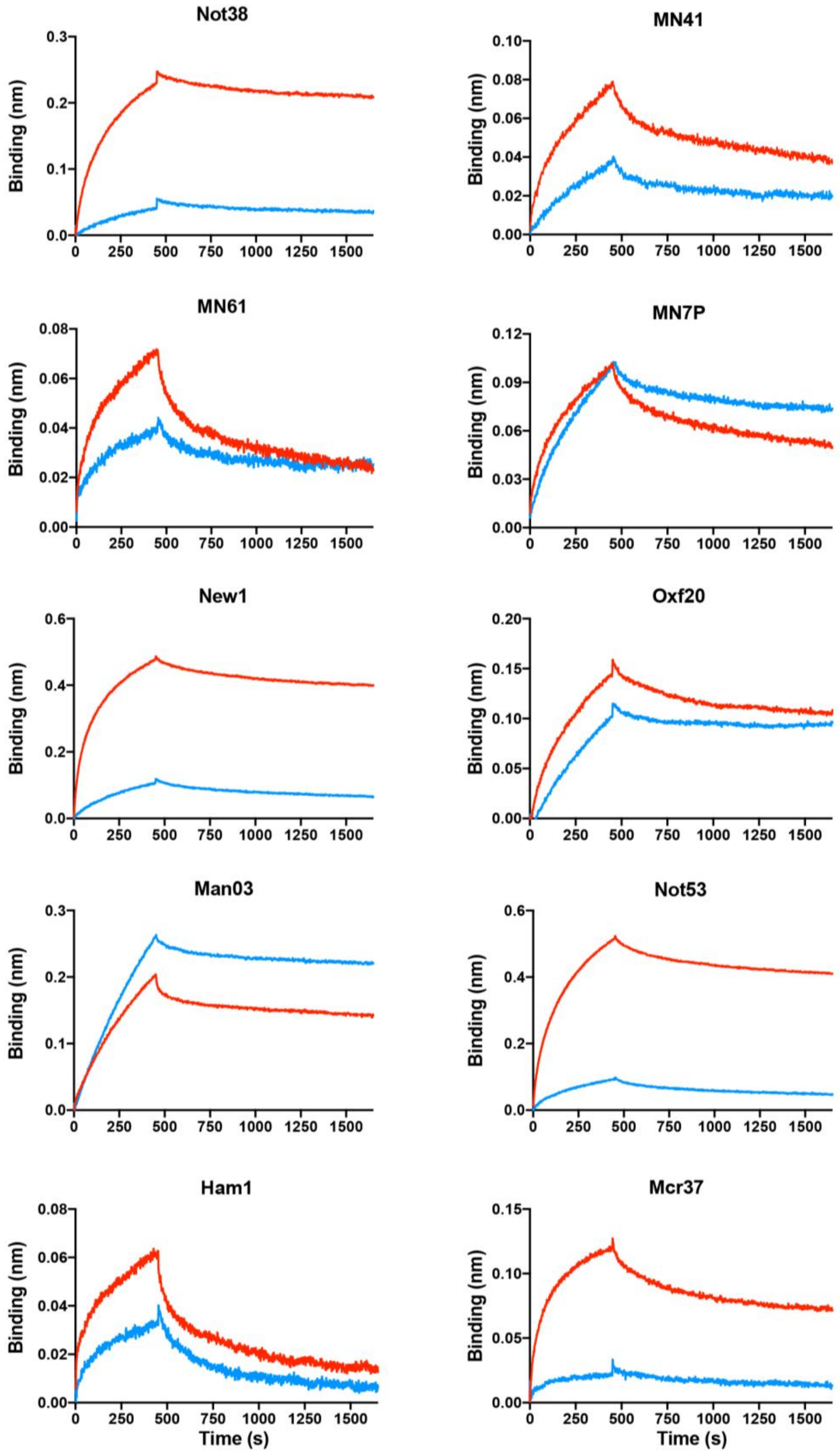

Fig.9 – Specificity of affinity purified anti-PLA2R to P28mer and T28mer

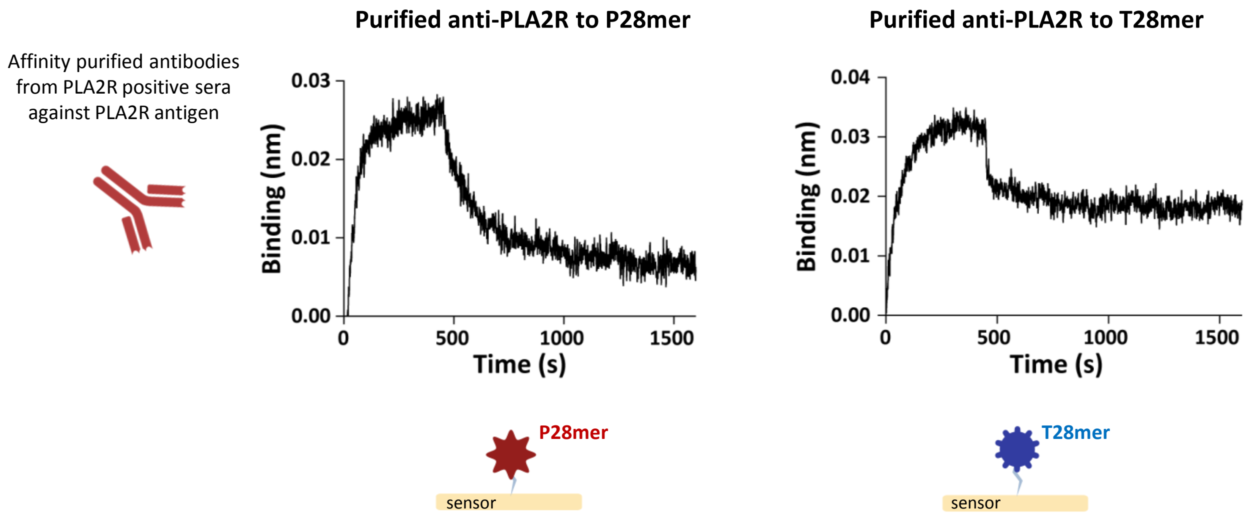

BLI sensorgrams of typical binding response of affinity purified anti-PLA2R from positive sera to either immobilized P28mer (left) or T28mer (right).

Fig.10 – Specificity of Moab 20-2-6 to P28mer and T28mer. Slot blot analysis of unrelated mouse monoclonal

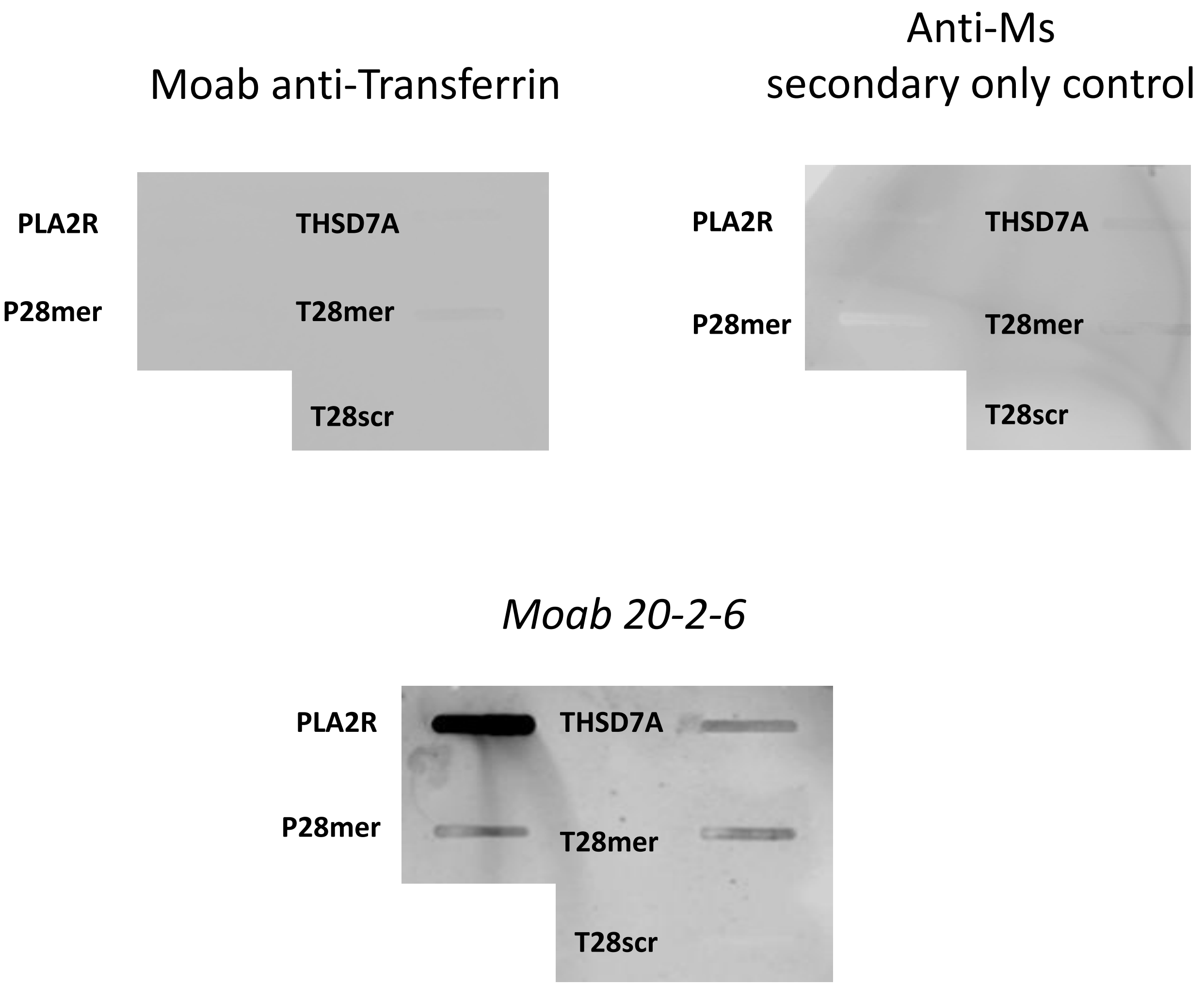

Slot blot images of PLA2R, THSD7A, P28mer and T28mer under non-reducing conditions incubated with an unrelated mouse monoclonal raised against transferrin receptor (left) or incubated with an Alexa Fluor® 680-AffiniPure Anti-Mouse IgG (right). Each slot was loaded with 1µg of protein/peptide and the membrane incubated with antibodies diluted 1:5000.
The bottom panel shows reactivity of Moab 20-2-6 to both peptides P28mer and T28mer.
